# Neurologic Care of COVID-19 in Children

**DOI:** 10.3389/fneur.2020.613832

**Published:** 2021-02-18

**Authors:** Susana Boronat

**Affiliations:** Pediatric Neurology, Hospital de la Santa Creu i Sant Pau, Barcelona, Spain

**Keywords:** SARS-CoV-2, seizures, epilepsy, neurocognitive, behavior, stress, child abuse

## Abstract

Most children with SARS-CoV-2 infection have relatively mild clinical symptoms without fever or pneumonia, although severe cases with multiple-organ failure have been reported. Neurological symptoms, which have been mainly reported in adults, are very rare in children. This article will review 2 different aspects of neurological involvement related to this infection in children. In the first part, we will review the neurological abnormalities reported in children caused by this viral infection. Adults frequently report muscle pain, headache, anosmia, dysgeusia, and occasionally more severe central or peripheral nervous system damage. Neurological involvement seems infrequent in children, although some cases have been reported. In the second part, we will discuss the COVID-19 pandemic impact on the healthcare system of some countries, causing collateral damage to general pediatric care and in particular to those children affected with chronic diseases, mainly neurological conditions, including autism, intellectual disability, attention deficit and hyperactivity disorder (ADHD), neuromuscular disorders, cerebral palsy, and epilepsy, and patients needing neurosurgical procedures.

## Introduction

The SARS-CoV-2 incubation period is usually 3–7 days, ranging from 1 to 14 days, and patients usually recover within 2 weeks. Typical clinical manifestations are dry cough and fever. Children are more frequently asymptomatic or they present with mild symptoms. Very rarely, severe pediatric cases may progress to acute respiratory failure and multiple-organ failure. The pediatric inflammatory multisystem syndrome, also referred to as Kawasaki-like SARS-CoV-2 syndrome, is a severe clinical picture that has been reported in children and shows persistent fever with multisystem organ involvement along with elevated inflammatory markers. It seems to appear in a late phase, as positive immunoglobulin G tests for SARS-CoV-2 have been reported in many cases (1–3).

In Table 1, we summarize the main topics of this article, including the main clinical neurological manifestations in children, current knowledge about treatment, and consequences of the COVID19 pandemic to children with neurological diseases.

**Table 1:** Summary.

Neurological involvement in children related to infection with SARS-CoV-2	• Encephalopathy • Inflammatory central nervous system lesions • Seizures • Stroke • Hyposmia and hypogeusia • Muscular involvement • Guillain-Barré syndrome • Other rare manifestations
Current treatment recommendations for children with SARS-CoV-2 infection	
Consequences of the COVID19 pandemic to children with neurological diseases	• Cerebral palsy • Neuromuscular disorders • Migraines • Neurosurgical conditions • Epilepsy • Behavioral problems • Autism and intellectual disability • Attention deficit and hyperactivity disorder • Psychiatric disorders

## Neurological Manifestations Related to SARS-CoV-2 Infection

Adults frequently report muscle pain, headache, anosmia, and dysgeusia and occasionally more severe central or peripheral nervous system damage. Within 214 patients from Wuhan reported by Mao and colleagues, 78 (36%) had neurological involvement, including neuralgic pain, myopathy, altered states of consciousness, and stroke. These symptoms were more frequent in severe cases (46 vs. 30% in non-severe) (4).

A recent meta-analysis including 41 articles showed a wide spectrum of neurological manifestations due to SARS-CoV-2 infection. The most frequent neurological symptoms included anosmia (36–86%) and dysgeusia (33–89%). More severe cases showed inflammation of the central nervous system, stroke, or Guillain–Barré syndrome (5).

## Neurological Involvement in Children Related to Infection With SARS-CoV-2

Twenty-one studies (14 about non-specific and 7 about specific neurological involvement) have been included in a systematic review of neurological manifestations in children published in September 2020. Out of 3,707 children, 581 (16%) had non-specific neurological manifestations, such as headache and fatigue, and 42 (1%) had specific neurological involvement including encephalopathy (25), meningeal signs (17), and/or seizures (12), which were more common in children with severe illness. Very rarely, Guillain–Barré syndrome, cranial nerve palsies, or intracranial hemorrhage were reported (6).

Next, I will review the current literature about specific neurological abnormalities in children infected with SARS-CoV-2.

### Encephalopathy

Encephalopathy of variable severity is the predominant definite neurological complication in children, usually in the setting of COVID-19 pediatric multisystem inflammatory syndrome. In these cases, aside from the infection process, septic shock or hypoxia may also play a causative role in some of these encephalopathic patients (6).

In one study, within 27 children with pediatric multisystem inflammatory syndrome related to SARS-CoV-2 infection, 4 (15%) had encephalopathy. Cerebellar and brainstem signs and muscle weakness were also detected. All patients improved and 2 showed total recovery. Interestingly, on the brain MRI, the splenium of the corpus callosum showed signal changes in all 4 patients. Other studies, including cerebrospinal fluid cells and PCR for SARS-CoV-2, oligoclonal band test or CNS autoantibodies were negative in all patients. Slow background activity was detected in the 3 patients who had EEG studies, and mild myopathic and neuropathic changes were seen in all 3 patients who were assessed by neurophysiologic studies (7). A previously healthy 16-year-old positive for SARS-CoV-2 developed seizures, encephalopathy, and syndrome of inappropriate anti-diuretic hormone (SIADH). Her CSF testing was negative for SARS-CoV-2. Although a thorough workup revealed no other infectious or autoimmune pathologies, MR imaging was not obtained (8). A lethargic 4-year-old girl with multisystem inflammatory syndrome associated with COVID19 showed aseptic meningitis and cytokine storm an low levels of BDNF (9).

### Inflammatory Central Nervous System Lesions

The SARS-CoV-2-related pediatric multisystem inflammatory syndrome is characterized by a Kawasaki-like illness with persistent fever and multisystem organ involvement, including cardiac dysfunction, with elevated inflammatory markers. Consistent with other cases with encephalopathy showing slow EEG background activity and lesions in the corpus callosum (7, 10), a 2-year-old presenting with altered mental status was found also to have a moderate background slowing in the EEG, but in this case, the MRI lesions involved the bilateral lateral thalamic nuclei, showing restricted diffusion without T2 or FLAIR anomalies (11). These lesions may resemble an acute necrotizing encephalopathy (ANE), although without a hemorrhagic component in this case. ANE is a rare manifestation of some viral infections, such as influenza, leading to bilateral thalamic damage and has been related to intracranial cytokine storms, which result in blood–brain barrier breakdown. At the onset of his encephalopathy, CSF SARS-CoV-2 PCR was negative, but interleukin-6 was elevated and went down gradually as he improved. Although more frequent in children, ANE can also affect adults, and it has been reported in an adult patient with SARS-CoV-2 infection (12).

One case of acute disseminated encephalomyelitis has been reported in a 12-year-old girl with SARS-CoV-2 infection. Five days after the initial presentation with headache, rash, and fever, she had respiratory failure related to generalized motor weakness. MRI showed an extensive cervical myelopathy and restricted diffusion involving cerebral white matter, including the splenium of the corpus callosum (13). Another two children have been reported with lesions of the corpus callosum, affecting mainly splenium, in the context of SARS-CoV-2-related pediatric inflammatory multisystem syndrome (14). These cases suggest that the splenium of corpus callosum may be a specially vulnerable area for inflammatory lesions in the context of SARS-CoV-2 infection with CNS involvement.

Recently, an international study with children with encephalopathy related to SARS-CoV-2 infection and abnormal neuroimaging findings was performed. Data were reviewed by a child neurologist, a pediatric infectious diseases expert, and a central neuroradiology panel. Neurological disease related to SARS-CoV-2 infection was reviewed in 38 children from France (*n* = 13), the UK (*n* = 8), the USA (*n* = 5), Brazil (*n* = 4), Argentina (*n* = 4), India (*n* = 2), Peru (*n* = 1), and Saudi Arabia (*n* = 1). The most common imaging abnormalities were post-infectious immune-mediated acute disseminated encephalomyelitis changes of the brain (16 patients), myelitis (8 patients), and neural enhancement (13 patients). Corpus callosum splenial lesions (7 patients) and myositis (4 patients) were predominantly observed in children with multisystem inflammatory syndrome (15).

### Seizures

Pediatric seizures associated with SARS-CoV-2 infection are usually acute symptomatic seizures and mainly occur during febrile episodes (6).

Two cases of febrile status epilepticus (16, 17) and two cases of status epilepticus in the setting of multisystem inflammatory syndrome due to SARS-CoV-2 have been reported (18, 19). One of these cases showed an occipital intracerebral hemorrhage (18).

Seizures have been reported as early as the first months of life, with or without fever. A 6-week-old with positive SARS-CoV-2 PCR presented with fever, cough, and brief episodes of impaired responsiveness with upward gaze and stiffening of both legs. EEG showed intermittent delta rhythm. All the other exams, including brain MRI, were normal (20). One male infant aged 26 days with SARS-CoV-2 infection and fever had 2 episodes of generalized hypertonia. The EEG was normal (21). A 3-month-old girl was reported to present with non-febrile focal seizures with impaired consciousness during SARS-CoV-2 infection at days 6 and 9 from onset, with normal EEG and MRI. D-Dimer and ferritin levels were increased. In this case, whole-exome sequencing was performed, revealing a pathogenic frameshift mutation in the *PRRT2* gene in both the mother and the infant. The mother had two late infantile febrile convulsions with normal neurological development. The authors suggested that SARS-CoV-2 may trigger non-febrile seizures in infants with a genetic predisposition (22).

A child presenting as isolated afebrile seizure in the setting of SARS-CoV-2 infection without other symptoms has also been reported (23). An adolescent with respiratory symptoms and lethargy showed epileptic apneic episodes (24).

### Stroke

Although SARS-CoV-2 predisposes to stroke in adults (4, 5), it is not the case for children, as other risk factors for stroke in children are usually absent. Angiotensin-converting enzyme 2 (ACE-2), located in the brain vascular endothelium, acts as a receptor for the virus, so clotting and infarction related to this mechanism might also be possible in children. However, stroke in children due to SARS-CoV-2 infection is exceptional, so the presence in adults of other risk factors for stroke seems to be very important for pathogenicity (25).

Coinfection by SARS-CoV-2 and tuberculous meningitis in a child was complicated by cerebral sinus venous thrombosis and arterial ischemic stroke, with elevated D-dimer, fibrinogen, and ferritin levels, suggesting that both microorganisms induced an important inflammatory prothrombotic situation leading to these complications (26).

One case with multisystem inflammatory syndrome that presented with status epilepticus showed an occipital intracerebral hemorrhage (18).

A previously healthy 14-year-old boy suffered an infarct involving middle and anterior right cerebral arteries. He developed arrhythmia with refractory shock, requiring extracorporeal life support, and died from the cerebral infarct. Postmortem microbiology was positive for SARS-CoV-2 (27).

A previously healthy 13-year-old girl who presented with loss of consciousness and was SARS-CoV-2 positive and had a ruptured pseudoaneurysm of the left middle cerebral artery (28).

Other stroke events have been associated with multisystem inflammatory syndrome in pediatric patients. A previously healthy 5-year-old who developed cardiogenic shock and required extracorporeal membrane oxygen had a right middle cerebral artery infarction, cerebral edema, and diffuse contralateral subarachnoid hemorrhage and died. A 2-month-old requiring extracorporeal membrane oxygen with refractory seizures developed multiple hemorrhagic infarctions. Although he tested negative for SARS-CoV-2 antibodies, his clinical presentation was suspicious for SARS-CoV-2 infection with elevated IL-6 levels (29).

Two children of 8 and 16 years of age suffered arterial ischemic strokes due to acute intracranial large vessel occlusion within 3–4 weeks of SARS-CoV-2 infection. One case presented with bilateral middle cerebral artery strokes and the other with multiple-organ system dysfunction. Neither patient fulfilled criteria for multisystem inflammatory syndrome in children (MIS-C) given absence of fever. These data suggest that systemic post-infectious arteritis with cerebrovascular involvement may complicate SARS-CoV-2 infection (30).

### Hyposmia and Hypogeusia

Anosmia/hyposmia and dysgeusia have been reported in 30–80% cases of SARS-CoV-2 in the adult population. As the olfactory neuroepithelium expresses ACE-2, a direct invasion by the virus causing inflammatory changes locally would lead to hyposmia, usually showing in the early stages of the disease (6). In children, hyposmia with or without hypogeusia was detected in 17 out of 27 (62%) cases (31). One child presented with isolated anosmia (32) and three adolescents showed these isolated symptoms in the absence of other symptomatology (33).

### Muscular Involvement

In a comparison between 7 children and 25 adults, while 13 adults (52%) complained of muscle pain, none of the children did. However, children showed elevation of creatine kinase levels more frequently than adults (57 vs. 4%) (34).

### Guillain–Barré Syndrome

Two cases of Guillain–Barré syndrome have been reported in children. An 11-year-old boy showed an acute inflammatory demyelinating polyneuropathy variant (35) and a 15-year-old boy presented with an acute motor axonal neuropathy (36). Both showed a favorable response to intravenous immunoglobulin.

### Cranial Polyneuropathy

At day 21 of a hematopoietic stem cell transplantation, a 6-year-old patient with cerebral vasculopathy due to sickle cell anemia presented with dysfunction of cranial nerves V, VII, and IX. Inflammatory anomalies were detected in these nerves in the brain MRI (37).

### Orbital Cellulitis With Intracranial Extension

Two adolescents with sinusitis and orbital cellulitis were reported. One had a hemorrhagic abscess, and the other an intracranial epidural abscess, with meningeal enhancement or extension in both cases (38).

### Current Treatment Recommendations for Children With SARS-CoV-2 Infection

General management of children with SARS-CoV-2 infection is based on supportive therapy.

Based on findings from the RECOVERY trial, corticosteroids are recommended in adult patients who are mechanically ventilated or who require supplemental oxygen. Because only a few pediatric patients were included in this trial, it is difficult to extend these recommendations for children. Dexamethasone may be used in pediatric patients requiring mechanical ventilation, but it is generally not recommended for those who require only minimal oxygen support (39) (Coronavirus disease 19 NIH Treatment Guidelines).

In a trial of adults hospitalized with SARS-CoV-2, remdesivir, a viral RNA–dependent RNA polymerase inhibitor, was shown to decrease time to recovery, but the effects are not known in the pediatric population (40) A phase 2/3 trial of remdesivir was initiated in June 2020 for children (ClinicalTrials.gov ID: NCT04431453). Convalescent plasma, which has recently been approved by the FDA for emergency use, has shown some efficacy in reducing mortality in critically ill patients (41) A phase 1 clinical trial of convalescent plasma in children is ongoing (ClinicalTrials.gov ID: NCT04377672).

There are insufficient data in children to recommend either for or against the use of other treatments that are currently under investigation (Coronavirus disease 19 NIH Treatment Guidelines).

Given the current scarce evidence of these treatments in pediatric patients, the standard management of neurologic complications, including ischemic stroke, seizures, and inflammatory lesions, is recommended at this point (39).

## Consequences of the Covid19 Pandemic Regarding Children Health Care

The COVID-19 pandemic has become an enormous challenge for many healthcare systems, mainly in the management of chronic patients. Children with neurological conditions are especially vulnerable to this situation.

### Delayed Access to Medical Care

Measures introduced by governments to try to delay the spread of infection have impaired the access to emergency departments or primary care facilities for illnesses not related to SARS-CoV-2 infection. Evidence from previous epidemics found that hospital avoidance during outbreaks of MERS and SARS was common, but emergency departments returned early to normality following these pandemics, as they lasted 2–3 months. This is not the case scenario for the COVID-19 pandemic, with still many months of pandemic ahead of us (42). This expected prolonged reduced access to health care will be damaging to children, mainly neurologically affected children with special needs, including cerebral palsy or epilepsy.

Lazzerini et al. reported some consequences from delayed access to medical care during COVID-19 pandemic in Italy during a week in March 2020. Twelve cases of delayed access to hospital care leading to severe complications or death were detected. These complications included mainly diabetic ketoacidosis, sepsis, seizures, and bowel obstruction. Half of the children were admitted to an ICU and four died. In all cases, parents reported avoiding accessing hospital because of fear of infection with SARS-CoV-2 (43).

Children with previous neurological conditions, including cerebral palsy or severe intellectual disability or autism, may be especially vulnerable, mainly because of their inability to communicate and because their parents are used to deal at home with their chronic conditions. Two children with cerebral palsy died in the ambulance on the way to the hospital, one after 10 days of fever and the other after 3 days of bloody stools. A child with intellectual disability due to Mowat–Wilson syndrome, in dialysis for chronic renal insufficiency, arrived at the hospital after 3 days of hypoactivity and died 4 days later in the ICU (43). This data demonstrates that children with neurological disorders are specially fragile in this situation, as 3 of the 4 deaths affected patients with neurological conditions.

### Changes in Modalities of Care

The COVID-19 pandemic has prompted to introduce rapidly telemedicine as one of the main modalities for patient clinic follow-ups. Visits by e-mail, telephone, or teleconference were quickly established at many hospital and primary care facilities in order to limit the spread of the virus. In some countries, including Spain, COVID-19 pandemic has accelerated the implantation of telemedicine in clinical care and it is planned to add this new modality to classical practice, during and once the pandemic is over.

### Impact on Mental Health

Children are at risk of suffering the consequences of parental affected mental health driven by stress related to the COVID-19 pandemic. Also, children themselves may have problems coping with the situation, leading to hyperactivity, challenging behavior, depression, and anxiety. Children with normal development may overcome the situation only with supportive intervention, but it may be more challenging in children with previous behavioral or psychiatric problems.

An Italian study found increased anxiety in a study of 148 healthy adolescents. The items concerning breathing difficulties and sleep disorder were the most affected (44).

## Consequences to Patients With Previous Neurological Diseases

### Cerebral Palsy

Patients with cerebral palsy, who usually are prone to lung problems, may be at risk for more severe respiratory symptoms due to SARS-CoV-2 infection, requiring more frequently ICU admissions. Also, they are specially at risk of suffering consequences for delayed access to the emergency department in case of an acute illness. Also, limited access to chronic care programs, including physical therapy, botulinum toxin or chronic surveillance, has been problematic for these children. For example, orthopedic follow-ups in children with cerebral palsy try to identify early hip displacement in order to establish treatment at the right time, before the hip becomes painful. The COVID-19 pandemic has led to the cancellation of clinical appointments and elective surgeries at many hospitals, worsening the standard of care that these patients need (45).

### Neuromuscular Disorders

Patients with neuromuscular disorders, including spinal muscular atrophy or muscular dystrophies, are especially at risk for respiratory complications in the setting of SARS-CoV-2. Specific consensus recommendations have been published to guide clinicians through these times of continuous adaptation of clinical care to the special needs of these patients (46, 47).

### Migraines

Patients with migraine are specifically vulnerable to this situation. Disruption of sleep and dietary habits, high psychosocial stress and social isolation, may lead to worsening of headaches. A survey of 1,018 adult patients showed an increase in migraine frequency and severity, and more transformation to chronic migraine. The authors identified some risk factors for migraines worsening, including difficult access to their neurologist, chronic treatment abandonment, disruption of sleep and dietary schedules, comorbidity with anxiety and depression, and working during the pandemic (48). Two Italian studies showed a general improvement in children and adolescents with primary headaches and migraines during the lockdown. Reduction in school-related stress seems to be the main factor (49, 50). In a study of children with migraine in India during the pandemic, around 90% of caregivers were satisfied with the efficacy of telephonic consultations (51).

### Neurosurgical Conditions

In general, urgent neurosurgery, for example, in cases of shunt malfunction, has not been delayed. It has been successfully performed during the pandemic with adequate measures of security in the operating room (52).

### Epilepsy

The COVID-19 pandemic has an important impact in care of people with epilepsy.

A group of international epilepsy experts published a consensus statement on June 2020 in order to provide clinical guidelines for epileptic patients (53).

This situation poses a risk to good epilepsy control, as patients may be unable to get the proper information to adjust drugs or dosages, leading to self-prescription or stop of medications without physician advice.

The Pediatric Epilepsy Research Consortium and the Child Neurology Society collaborated to issue recommendations for diagnosis, treatment, and follow-up of infantile spasms in this special time (54) advocating for oral therapies as initial treatment and use of telemedicine. Also, a guideline for Chinese epileptic children has been published. The authors emphasize the use of telemedicine for care and provide a useful guideline of medications for SARS-CoV-2 that might have interactions with antiepileptic drugs (55).

A Spanish survey in April 2020 assessed the impact of the COVID-19 pandemic in patients with genetic developmental epileptic encephalopathies. Within 277 responders, 39 (14%) reported seizure frequency increase and 87 (30%) cases showed behavioral deterioration during the lockdown. In addition, nine patients experienced some degree of neurological regression and there was one case of status epilepticus. The main factors contributing to seizure or behavioral changes were inability to get the proper access to medical care, including difficulties reaching their neurologist, avoidance of seeking medical advice in the emergency department due to fear of infection, loss of regular stimulation and physical therapies, cancelation of essential medical appointments, and difficulties finding their antiepileptic drugs at a pharmacy. The oversaturation of the healthcare system was especially important in Madrid and Barcelona, the Spanish regions with the highest incidence of COVID-19. Also, new-onset depression or anxiety in caregivers, living in small homes without a terrace or yard, and economic problems were factors leading to worsening of these patients (56).

### Behavioral Issues

Children with behavioral problems treated with regular psychological therapy are at high risk of worsening during the COVID-19 pandemic, due to isolation from peers, family stress, and imposed changes in routine. Some countries have implemented online therapy programs that allow to maintain physical distance and provide emotional support at the same time. Mental health of parents must be assessed, as they have direct impact on their child's behavior (57).

### Autism and Intellectual Disability

Lifestyle changes imposed by the COVID-19 pandemic are hard especially for those with intellectual disability and/or autism, as they have problems with understanding the situation, communicating, and expressing their feelings, which may lead to behavioral problems, including self-aggressions. Because of their need of predictable routines, and preference for certain activities, they may feel frustrated with schedule changes. Their progress is at risk, and they may even show regression of some skills. Scheduling specific times for wake and sleep, exercise, schoolwork, and meals is important for all children and even more in individuals with neurodevelopmental problems (58).

### Attention Deficit and Hyperactivity Disorder (ADHD)

Children with ADHD are also at risk in this situation. The loss of daily routine will worsen ADHD symptoms. The psychological stress lived by parents during the COVID-19 pandemic may exacerbate the children's symptoms As expected, a study among Chinese school-aged children with ADHD showed that symptoms were significantly worse during the COVID-19 outbreak compared to normal state (59). The European ADHD Guidelines Group (EAGG) has addressed these issues and developed a guideline for the management of ADHD through the pandemic (60).

### Psychiatric Disorders

Individuals with mental health conditions are especially vulnerable to worsening of their symptoms during this pandemic. Some individuals manifest these mental issues as somatic complains, such as pain or breathing difficulties. Somatic symptom disorders may peak in this setting, as demonstrated by a case report of a 16-year-old adolescent, who despite testing negative for SARS-CoV-2, had an obsessive preoccupation about being infected and required an admission to the Psychiatric Unit, showing a rapid response to antidepressant and antipsychotic medications (61).

## Discussion

We have reviewed the neurological clinical findings in children infected with SARS-CoV-2 published at this time, but it is expected to gather more knowledge in the future, as the COVID-19 pandemic evolves.

Neurological complications of SARS-CoV-2 infection in children are much less frequent than in adult population. Around 16% of children show non-specific neurological symptoms as headache, while more severe and specific neurological complications occur only around 1% of cases.

Children with SARS-CoV-2 multisystem inflammatory syndrome are especially at risk for neurological complications. Encephalopathy is the more frequent abnormality, sometimes with symptomatic seizures, and it is usually transitory, without long-term neurological sequelae. In some patients, EEG and brain MRI show transient abnormalities, which usually resolve with time (6, 7).

The COVID-19 pandemic has supposed a struggle for healthcare systems around the world. Children with neurological conditions and their families are especially vulnerable.

Current data support the beneficial role of telemedicine in the care of these patients. Parents of children with neurological conditions need support, and they have to be encouraged to seek medical attention when needed and go to the emergency departments to avoid delay in diagnosis and treatment of potentially life-threatening conditions, such as infections, appendicitis, and shunt malfunctions. Delays in getting the adequate medical attention and other problems with neurologically affected children during the pandemic leading to severe problems and even death have been clearly documented.

Risk of abuse and neglect of children during the COVID-19 outbreak may peak due to parents' stress related to a mix of factors, including fear of illness, working from home while caring for children, financial insecurity, and lack of free time outdoors. With schools closed and families isolated in their homes, children are separated from teachers and healthcare workers, which are an important source of protection as they may detect and report suspected abuse (62, 63). A rise in pediatric blunt trauma, including skull and long bones fractures, face contusions, and intracranial hemorrhages, was reported in a hospital during the COVID-19 pandemic (64). This difficult time for some families, struggling with psychological stress and financial problems, must raise high suspicion for child abuse in some cases, mainly in behavior-challenging individuals such as those with ADHD, autism, and neurodevelopmental disorders (59, 63).

The healthcare system of every country has the obligation to assure adequate care for these children until the end of the pandemic.

## Author Contributions

The author confirms being the sole contributor of this work and has approved it for publication.

## Conflict of Interest

The author declares that the research was conducted in the absence of any commercial or financial relationships that could be construed as a potential conflict of interest.
